# Molecular contrast agents for post-infarct cardiac remodelling: a contemporary review

**DOI:** 10.1093/ehjimp/qyag019

**Published:** 2026-01-29

**Authors:** Pak Yin Lam, William D Watson, Ziad Mallat, Jason Tarkin

**Affiliations:** GKT School of Medical Education, King’s College London, 1/F Henriette Raphael Building, Guy's Campus, London SE1 1UL, UK; Section of Cardiorespiratory Medicine, University of Cambridge, Victor Phillip Dahdaleh Heart & Lung Research Institute, Cambridge, UK; Section of Cardiorespiratory Medicine, University of Cambridge, Victor Phillip Dahdaleh Heart & Lung Research Institute, Cambridge, UK; Section of Cardiorespiratory Medicine, University of Cambridge, Victor Phillip Dahdaleh Heart & Lung Research Institute, Cambridge, UK; Section of Cardiorespiratory Medicine, University of Cambridge, Victor Phillip Dahdaleh Heart & Lung Research Institute, Cambridge, UK

**Keywords:** myocardial infarction, cardiac remodelling, molecular imaging, PET, MRI

## Abstract

Myocardial infarction (MI) is a leading cause of mortality, affecting 3.8% of the global population under 60 years of age, and 9.5% over 60 years. Adverse post-MI remodelling is the main contributor to heart failure, conferring a three- to six-fold increase in risk of premature death. Current imaging modalities, such as nuclear imaging and magnetic resonance imaging, provide structural and functional information of the heart and identify extracellular expansion for indirect estimation of infarct size. However, they do not characterize the exact biological mechanisms involved, nor the staging of disease progression. Molecular imaging aims to visualize the underlying processes of remodelling at a molecular and cellular level, aiding more accurate prognosis and targeted treatment. Molecular tracers, such as radioisotopes and paramagnetic nanoparticles, are modified to adhere to specific molecules or cells involved in inflammation, angiogenesis, and scar formation, which can be visualized and quantitatively assessed using positron-emission tomography and magnetic resonance imaging. This article provides an overview of the pathophysiology of adverse remodelling, the latest molecular contrast agents under development, and future directions for integration into clinical practice.

## Graphical Abstract

**Graphical Abstract:**
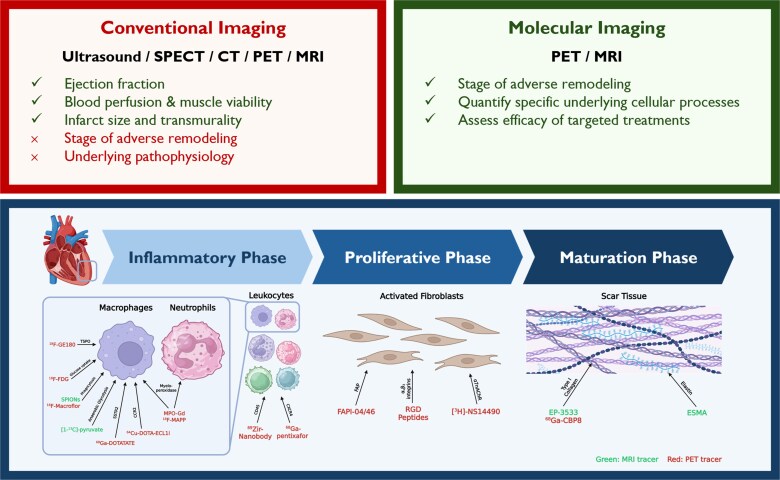

## Introduction

Cardiovascular disease (CVD) is the most common cause of death worldwide. In 2019, it was attributed to over 30% of global deaths.^1^ Coronary artery disease, especially myocardial infarction (MI), remains a leading cause of mortality, affecting 3.8% of the global population under 60 years of age, and 9.5% over 60 years.^2^

MI arises from obstruction of blood flow to the myocardium, often from a ruptured atherosclerotic plaque, causing thrombosis, leading to cardiomyocyte death and subsequent necrosis of the infarcted area. The heart subsequently undergoes structural and functional changes in an attempt to repair the infarct. This is known as cardiac remodelling, and can be divided into three distinct but overlapping phases: inflammatory (hours to days), proliferative (days to weeks), and maturation (months to years).^3^ Imbalance or overactivity of these processes hinders myocardial recovery, causing arrhythmias, ventricular dilatation, and contractile dysfunction, increasing the likelihood of heart failure (HF). To date, MI is the most common cause of HF, affecting 20–30% of patients within 1 year of discharge,^4^ and conferring three- to six-fold increase in premature death risk. Survivors of HF also experience a reduction in quality of life, such as long-term limitations in physical activity, sleep disturbances, and inability to work.^5^ Moreover, the rise in HF incidence has imposed substantial financial costs; in the UK, it accounts for 2% of the National Health Service budget.

Identification of adverse remodelling is critical for minimizing the risk of post-MI HF by improving prognosis and timely treatment. Current imaging modalities, such as nuclear imaging and magnetic resonance imaging (MRI), provide structural and functional information of the heart and an estimate of infarct size. However, these assessments are non-specific and do not indicate the stage of adverse remodelling. New molecular imaging techniques aim to overcome these limitations by targeting specific biological processes of remodelling. This article reviews recent progress in the use of molecular imaging for post-infarct myocardial remodelling and future directions for translation to clinical practice.

## Pathophysiology of post-MI remodelling

### Inflammatory phase

Within the first few hours to days, cardiomyocyte injury initiates an intense inflammatory response. The inflammation may be further amplified upon revascularization, due to myocardial reperfusion injury. Damage-associated molecular patterns, released by necrotic cardiomyocytes, stimulate the production of pro-inflammatory cytokines. These recruit leukocytes to the infarct zone, where they clear up dead cells and extracellular matrix (ECM) debris.^6^ Neutrophils are recruited the earliest, then macrophages and lymphocytes follow. Cytokines also activate matrix metalloproteinases, which degrade the intermyocyte collagen network in the ECM, causing the infarct zone to expand.

Between Days 4 and 7, the inflammatory response subsides. Neutrophil apoptosis plays a key role in this reparative process, releasing pro-resolving mediators such as lactoferrin, and stimulating macrophage production of lipoxins, interleukin (IL)-10, and transforming growth factor-β (TGF-β).^6^ These signals inhibit cytokine production and leukocyte recruitment, and stimulate regulatory T-cell activity. Furthermore, they induce macrophage switching to anti-inflammatory phenotypes. Timely resolution of the inflammatory phase is critical for subsequent wound healing. Excessive or prolonged inflammation may lead to impaired scar formation and uncontrolled infarct expansion towards the remote myocardium, resulting in adverse remodelling and wall dilatation.

### Proliferative phase

As the inflammation subsides, the injured myocardium begins to heal. Growth factors such as TGF-β, platelet-derived growth factors, and fibroblast growth factors stimulate transdifferentiation of interstitial fibroblasts and endothelial cells into synthetic myofibroblasts.^7^ They express contractile proteins such as α-smooth muscle actin, stimulate angiogenesis, and lay down a network of elastin and collagen fibres in the ECM. This is supported by activation of the renin–angiotensin–aldosterone system (RAAS), particularly angiotensin II and aldosterone, which stimulate fibroblast proliferation and ECM protein synthesis through the AT1 receptor, Kirsten Ras-A, and MAPK1/2 pathways.

### Maturation phase

Over the next months to years, the ECM is continuously being remodelled. Collagen and elastin fibres interweave and are strengthened by lysyl oxidase enzymes, establishing a framework for scar formation.^8^ Collagen, mainly types I and III, provides structural integrity to the scar, while elastin maintains elasticity and recoil of the myocardial wall.

Together, collagen and elastin work hand-in-hand to maintain the tensile strength of the scar. The extent of cross-linking is mediated by a delicate balance between the level of inflammation and mechanical strains on the heart. A weak, poorly structured scar is subject to thinning and rupture, while excessive fibrosis causes myocardial stiffening. Both pathologies result in irreversible contractile dysfunction and reduction in cardiac output.^9^

Additionally, the remote healthy myocardium undergoes hypertrophy to compensate the need for circulation. This is stimulated by myocardial stretch, the sympathetic nervous system, RAAS, and growth factors. Although hypertrophy helps maintain stroke volume temporarily, long-term overstretching may eventually lead to chronic dilatation.^10^ Ventricles lose their ellipsoid shape and become more spherical, with raised end-systolic and end-diastolic volumes and reduction in ejection fraction.

## Molecular imaging

### Why molecular imaging?

Current imaging modalities for post-MI remodelling focus on providing structural and functional information of the myocardium. Conventional imaging techniques such as echocardiography and MRI visualize changes, for example, ventricular dilatation, wall thinning, and ejection fraction. MRI with late gadolinium enhancement (LGE-MRI) can also provide information on the extent and transmurality of the infarct. Nuclear imaging with conventional tracers such as Technetium-99m sestamibi for single-proton emission computer tomography (SPECT), as well as rubidium and fludeoxyglucose (FDG) for positron emission tomography (PET), can be used to evaluate for perfusion abnormalities and myocyte viability.

However, these techniques only provide indirect insight into the consequences of adverse remodelling. For example, LGE-MRI only detects extracellular expansion in the myocardial wall. This is a non-specific finding that can occur at any stage of remodelling, and may also be seen in other conditions such as amyloidosis, sarcoidosis, or myocarditis. The lack of specificity for the underlying biological processes of remodelling hinders its prognostic utility in predicting long-term adverse cardiac events. While systemic pharmacological treatments post-MI HF such angiotensin converting enzyme (ACE) inhibitors, angiotensin II receptor blockers, beta-blockers, aldosterone, angiotensin and neprilysin receptor inhibitors, and finally, sodium-glucose transport protein 2 inhibitors help prevent adverse myocardial remodelling, the emergence of newer therapies such as those targeted at inflammation means that imaging will be essential to guide their use and predict treatment effectiveness on individual patients.

Molecular imaging provides direct visualization of the mechanisms behind remodelling *in vivo* at a molecular and cellular level, offering a deeper understanding of the underlying pathophysiology, earlier identification of ongoing and potentially reversible processes, more accurate risk stratification, and potentially aid the development and monitoring of inflammation or fibrosis-targeted precision treatments. To date, a variety of molecular contrast agents have been proposed for each of the three phases of remodelling (*Table 1*).

**Table 1:** Molecular imaging targets for inflammatory, proliferative and maturation stages of adverse remodelling

Tracer	Imaging modality	Molecular and cellular targets	Summary of findings
*Inflammatory phase*			
SPIONs^11–13^	MRI	Macrophages	SPIONs localized specifically to CD68^+^ macrophages. Higher sensitivity than LGE-MRI. Uptake increased in infarct areas with reduced ventricular contractility in humans. Able to track longitudinal changes in macrophage activity over 3 months.
MPO-Gd^14^	MRI	Myeloperoxidase on neutrophils and macrophages	Stronger and more persistent enhancement than LGE-MRI in infarct regions. Able to monitor therapeutic effect of atorvastatin on MPO activity in mice.
^18^F-MAPP^15^	PET		Enables detection of both intracellular and extracellular MPO activation. Able to track therapeutic effect of MPO inhibitor PF-2999 in mice.
[1-^13^C]-pyruvate^16,17^	MRI	Anaerobic glycolysis in pro-inflammatory macrophages	Able to detect anti-inflammatory effect of glycolytic inhibitor 2-deoxyglucose in mice through reduced [1-^13^C]-lactate production. Able to identify viable myocardium through preserved ^13^C-bicarbonate production.
^18^F-FDG^18,19^	PET	Macrophages	Uptake increased in infarct areas verified by LGE-MRI. Inverse correlation between ^18^F-FDG uptake and long-term ventricular function. Able to quantify effect of NLRP3 inflammasome inhibitor MCC950 on macrophage and neutrophil infiltration in humans. Able to track resolution of inflammation with Evolocumab (PCSK9 inhibitor).
^68^Ga-DOTATATE^20,21^	PET	SST2 receptors on macrophages	Lower background signal compared to ^18^F-FDG-PET. Able to highlight residual macrophage activity in chronic infarction.
^18^F-Macroflor^22^	PET	Macrophages	Visualized macrophage accumulation in the infarct myocardium and atherosclerotic plaques of mice and rabbits. Combined multi-modal imaging with MPO-Gd MRI showed macrophage expansion and shift towards pro-reparative phenotypes.
^89^Zir-CD45 nanobody^23,24^	PET	CD45 on leukocytes	Able to monitor changes in inflammatory activity over time in mouse models of ARDS, IBD and GvHD. Higher specificity and localization compared to ^18^F-FDG-PET.
^68^Ga-pentixafor^25–28^	PET	CXCR4 on leukocytes	Strong concordance with CD45^+^ leukocytes, CD68^+^ macrophages and Ly6G^+^ granulocytes in mice. Able to quantify effect of AMD3100 (small-molecule antagonist) on CXCR4 activity over 7 days in mice. In humans, high uptake in the infarct regions correlated with reduced long-term cardiac function, and predicted risk of MACEs following acute MI. High uptake in the mediastinal lymph nodes correlated with reduced myocardial necrosis, indicating reparative T-cell activity.
^64^Cu-DOTA-ECL1i^29,30^	PET	CCR2 on macrophages	In MI patients, strong concordance with areas of reduced perfusion and ventricular motion verified by Tc-99m-SPECT/CT. Combined imaging with ^89^Zr-labelled CD11b nanotracer showed inverse correlation between myeloid cell infiltration and ventricular function.
^18^F-GE180^31^	PET	TSPO on macrophages and microglia	Higher uptake on pro-inflammatory macrophages than anti-inflammatory macrophages. Early uptake in the infarct was associated with adverse remodelling at 8 weeks. Over time, uptake shifted from initial infarct to the remote myocardium, indicating ongoing mitochondrial dysfunction and adverse remodelling. Concomitant uptake in brain tissue indicates persistent neuroinflammation. Able to monitor effect of ACE inhibitor treatment on reducing heart and brain inflammation.
*Proliferative phase*			
FAPI-04/46^32–34^	PET	Fibroblast activation protein on activated fibroblasts	High signal uptake associated with regions of low metabolic activity from ^18^F-FDG-PET, and subsequent ventricular dysfunction on follow-up MRI. Combined imaging with FAPI-04 and LGE-MRI can identify early and potentially reversible fibroblast-associated remodelling.
RGD peptides^35–38^	PET/SPECT	α_v_β_3_ integrins on activated fibroblasts	Able to monitor effect of ACE inhibitors and aldosterone antagonists on reducing excessive fibroblast activity. Early uptake associated with subsequent improvements in ventricular function in the long term.
[^3^H]-NS14490^39^	PET	ɑ7nAChR on fibroblasts	Uptake increased from 2 weeks post-MI, peaking at 4 weeks. Strong signal uptake associated with ɑ7nAChR expression and ECM production.
*Maturation phase*			
EP-3533^40^	MRI	Type I collagen	Localized specifically to collagen in scar tissue, verified by histological staining. Stronger and more persistent enhancement compared to Gd-DTPA.
^68^Ga-CBP8^41^	PET		Tested in mice and patients with idiopathic pulmonary fibrosis (IPF). Localized to collagen not only in areas of known fibrosis, but also in regions of active/recent collagen synthesis not visible in CT imaging.
ESMA^42,43^	MRI	Elastin	Stronger and more persistent enhancement compared to Gd-DTPA. Clear visualization of infarct scar up to 30 days post-MI.

### Leukocyte-targeting agents for the inflammatory phase

The inflammatory phase is initiated shortly after ischaemic injury, triggering waves of immune responses. Early inflammation is characterized by infiltration and accumulation of leukocytes such as neutrophils and macrophages, the extent of which plays a critical role in subsequent tissue healing and long-term adverse remodelling. Immunomodulatory therapies have been a recent focus for the prevention of post-MI HF by attenuating this acute inflammatory response. Notable treatments include canakimumab, an IL-1β targeting monoclonal antibody which reduced the incidence of MI, stroke and cardiovascular death by up to 13% in a randomized trial of over 10 000 patients with previous MI and high high-sensitivity C-reactive protein (hsCRP)^44^; and Anakinra, a human recombinant IL-1 receptor antagonist which significantly reduced all-cause mortality and new-onset HF in a pooled analysis of three randomized trials.^45^ Consequently, there is an ongoing demand for suitable biomarkers that can identify individuals most likely to benefit from these treatments, as well as quantify treatment response. While previous studies have used systemic biomarkers such as plasma hsCRP,^46^ they do not inform local inflammation processes within the myocardium. Leukocyte-targeting molecular biomarkers enable more specific imaging of myocardial inflammatory processes at the injury site.

#### Macrophage-targeting superparamagnetic iron oxide nanoparticles for MRI

**Figure 1:**
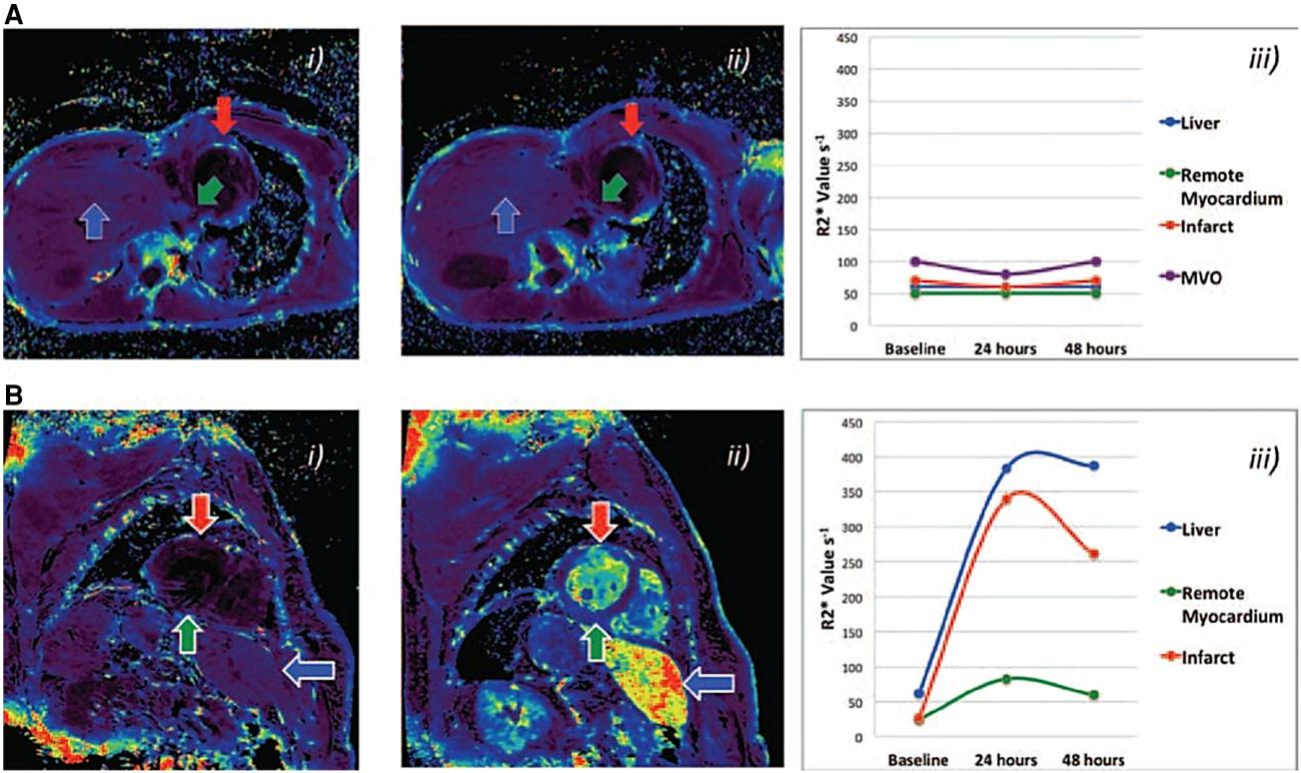
Visualizing macrophage-driven inflammatory activity using USPIONs. R2* colour maps in MI patients at i) baseline and ii) 24 h in *A*) control patients and *B*) patients who underwent USPION infusion. iii) Corresponding R2* values in the liver (blue), infarct region (red) and remote myocardium (green) in control patients vs. patients who received USPIONs. R2* increased in the liver and myocardium in patients who received USPIONs, but remained unchanged in control patients. Reproduced from Alam *et al.*, copyright permission obtained from Wolters Kluwer Health Inc. (CC-BY-NC).^11^

During acute inflammation, circulating monocytes migrate *en masse* to the infarct region, where they differentiate into macrophages and accumulate. Superparamagnetic iron oxide nanoparticles (SPIONs) are ideal for imaging macrophages. They consist of an iron oxide core (diameter 10–100 nm) surrounded by a polymer coating, which stabilizes the particle *in vivo*. In the early inflammatory phase, they extravasate into the infarct area and are phagocytosed by macrophages as foreign bodies into the cytoplasm. There, they shorten T2* relaxation, generating signal voids in T2*-weighted images. Being superparamagnetic, they have much higher sensitivity than gadolinium, making detection possible even at nanomolar concentrations. Following intravenous administration of the ultrasmall SPION (USPION) Ferumoxytol in MI patients (*n* = 16), a significant increase in R2* values was observed in the infarct within 24 h (*P* < 0.001) and 48 h (*P* < 0.05) (*Figure 1*).^11^ In another clinical study on 14 patients with acute ST-elevation MI (STEMI), Ferumoxytol led to substantial reduction in T2* values in the infarct within 24 h, while minimal change was observed in the skeletal muscle.^12^ Stirrat *et al.* further explored the utility of Ferumoxytol for tracking longitudinal inflammatory changes.^13^ Following repeated doses in 31 patients, uptake in the infarct peaked at Days 2–3, and persisted until Days 10–16 (*P* < 0.05). This was associated with myocardial tissue oedema on T2 imaging that peaked at Days 3–9, and remained increased up to 3 months (*P* < 0.01).

Despite success in early clinical studies, clinical adoption of SPIONs in myocardial imaging is not yet widespread. One major limitation is slow clearance rates. Because SPIONs are cleared from the blood pool via the reticuloendothelial system, patients are required to wait for up to 6–24 h before imaging, as opposed to several minutes for LGE-MRI. The substantial increase in waiting times diminishes its feasibility for clinical applications. Furthermore, long-term retention of iron oxide can cause chronic damage to the body due to oxidative stress.

#### Myeloperoxidase-targeting gadolinium agents for MRI

Myeloperoxidase (MPO) is an enzyme produced by neutrophils and macrophages that catalyses chloride oxidation to hypochlorous acid, generating various reactive oxygen species (ROS), for example, chlorine, tyrosyl radicals, and aldehydes. These ROS are cytotoxic and inhibit enzymes such as ATPase and cross-link proteins, impairing scar formation and cardiac contractility. Monitoring its distribution throughout the infarct region could therefore be a reliable indicator of adverse remodelling from an early stage.

5-Hydroxytryptamide, or MPO-gadolinium (MPO-Gd), targets MPO activity in the myocardium. Upon activation by MPO, it polymerizes into a bulkier molecule and shortens T1 relaxation, producing bright regions in T1-weighted imaging. In mice (*n* = 56), MPO-Gd generated an enhancement 4× higher (contrast-to-noise ratio 40.8 ± 10.4 vs. 10.5 ± 0.2, *P* < 0.05) and lasting twice as long in the infarct compared to gadolinium-diethylenetriamine penta-acetic acid (Gd-DTPA), the mainstream non-specific agent for LGE-MRI.^14^ Serial imaging further showed that MPO-Gd could monitor the anti-inflammatory effects of atorvastatin by tracking the attenuation of MPO activity over 24 h.

More recently, the same group developed an MPO-targeting PET imaging agent, ^18^F-MAPP.^15^ Because it does not rely on a metal centre or chelating backbone, unlike MPO-Gd, ^18^F-MAPP can cross cell membranes, enabling detection of intracellular MPO activity in addition to extracellular MPO-driven activation. In mice, ^18^F-MAPP was able to track the treatment effects of MPO inhibitor PF-2999, with signal changes corroborated by autoradiography and gamma-counting data.^47^ 50 mg/kg PF-2999 reduced myocardial ^18^F-MAPP uptake by 40%, which aligned with significant reductions in both intracellular and extracellular MPO activity on enzyme assays. To date, MPO-targeting agents have only been evaluated in pre-clinical models; further clinical studies are needed to establish their safety and effectiveness in humans.

#### ^13^C tracers for hyperpolarized MRI

Hyperpolarized MRI is a recently emergent technique that involves manipulating the magnetic properties of particles, using methods such as dynamic nuclear polarization, to improve signal-to-noise ratios up to several orders of magnitude compared to conventional MRI. Hyperpolarized metabolic molecules, such as ^13^C-pyruvate, can be used to visualize key cellular inflammatory pathways *in vivo* by detecting downstream metabolic products. During aerobic respiration, pyruvate is converted by pyruvate dehydrogenase into acetyl-coenzyme A and carbon dioxide, which rapidly equilibrates with bicarbonate. In contrast, pyruvate is converted to lactate during anaerobic glycolysis, a process upregulated in metabolically stressed or oxygen-deficient cells. During inflammation, pro-inflammatory macrophages shift towards anaerobic glycolysis, resulting in elevated lactate and reduced bicarbonate production. This makes ^13^C-pyruvate a useful tool for imaging inflammation and myocardial viability through the detection of ^13^C-lactate and/or ^13^C-bicarbonate. In mice, hyperpolarized MRI using [1-^13^C]-pyruvate showed strong [1-^13^C]-lactate signal in the infarct at Days 3 and 7 post-MI.^16^ Systemic administration of 2-deoxyglucose, a glycolytic inhibitor, significantly reduced ^13^C-lactate signal in the infarct zone within 3 days post-MI. This was associated with reduced inflammatory cytokine production and improved cardiac function at 3 months, demonstrating the potential of hyperpolarized MRI for assessing inflammation as well as monitoring responses to immunomodulatory treatments.

Subsequently, Apps *et al.* reported the use of hyperpolarized MRI for the first time in two MI patients, where they were able to identify viable parts of myocardium based on preserved ^13^C-bicarbonate signal, indicating ongoing aerobic metabolism.^17^ Mapping these viable segments may ultimately be useful for stratifying patients who would most likely benefit from revascularization.

#### ^18^F-FDG-PET

^18^F-fludeoxyglucose (^18^F-FDG), a radioactive glucose analogue, can be used to visualize cells with high metabolic activity with PET. Traditionally utilized with MRI to estimate infarct size by delineating areas of low myocardial viability, it was also trialled for targeting activated macrophages in a clinical study on 49 STEMI patients.^18^ Strong concordance was observed between FDG uptake and LGE-MRI, with inverse correlation to ventricular function at 6 months. Li *et al*. used ^18^F-FDG-PET to quantify the anti-inflammatory effects of MCC950, an NLRP3 inflammasome inhibitor, in MI mice.^19^ Lower uptake was associated with reduced macrophage and neutrophil infiltration, and improved myocardial viability. Similarly, Ziogos *et al.* in the recent EVACS (Evolocumab in Patients with Acute Myocardial Infarction) trials used ^18^F-FDG-PET to track the effects of proprotein convertase subtilisin/kexin 9 (PCSK9) inhibitor Evolocumab.^48^ Reduced uptake at 30 days post-MI was associated with improved resolution of inflammation and improved ventricular function at 6 months (SUV_mean_ vs. global longitudinal strain *r* = 0.25, *P* = 0.044).

However, the use of ^18^F-FDG for inflammation imaging is often ineffective, leading to non-diagnostic scans. It also requires prolonged fasting/dietary manipulation prior to imaging, to temporarily suppress background ^18^F-FDG uptake in cardiomyocytes as they switch to using fats for energy.

#### SSTR2-PET

**Figure 2:**
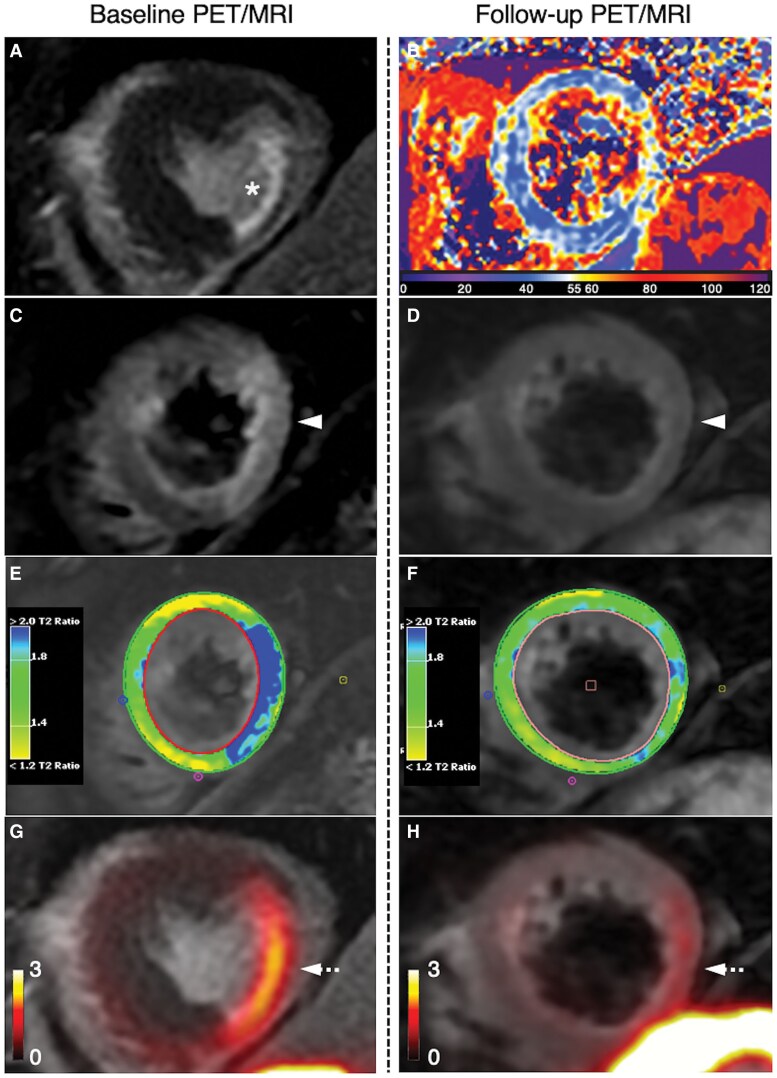
Tracking myocardial inflammation using ^68^Ga-DOTATATE PET/MRI at baseline vs. 3-month follow-up. Baseline PET/MRI (left): *A*) LGE-MRI showing subendocardial MI (white asterisk) in the mid inferolateral segment, with corresponding *C*) high signal (white arrowhead) on T2-weighted imaging, and *G*) increased 68Ga-DOTATATE uptake (white dashed arrow). *E*) Colour map shows myocardium-to-muscle T2 ratio. Follow-up PET/MRI (right): *B*) T2-mapping; *D*) resolution of oedema on T2-weighted imaging; *F*) myocardium-to-muscle T2 ratio; *H*) residual 68Ga-DOTATATE uptake indicating ongoing macrophage activity (white dashed arrow). Reproduced from Ćorović *et al.*, open access under Creative Commons CC-BY 4.0.^49^

To eliminate the need for dietary myocardial suppression, other macrophage-targeting PET tracers with lower background myocardial binding have been proposed.^20^ One example is ^68^Ga-DOTATATE, a somatostatin receptor (SSTR2) targeting agent. Originally used for neuroendocrine tumours, Tarkin *et al*. demonstrated their ability to bind to CD68^+^ macrophages within atherosclerotic plaques in humans (*n* = 42).^21^ Subsequent post hoc analysis of the study, including 12 MI patients, showed that ^68^Ga-DOTATATE could identify both active inflammation in recently infarcted myocardium, as well as chronic inflammation in old ischaemic areas, likely reflecting residual macrophage-driven inflammatory activity. The ability of ^68^Ga-DOTATATE to track areas of resolving myocardial inflammation has also been shown in a prospective longitudinal cohort study using PET/MRI (*Figure 2*).^49^

#### ^18^F-macroflor PET

^18^F-Macroflor is a polyglucose nanoparticle PET agent with a high avidity for macrophages. Similar to SPIONs for MRI, ^18^F-Macroflor is phagocytosed by macrophages as foreign bodies. It has successfully been used to visualize acute inflammatory macrophage activity in the myocardium of MI mice, as well as within the atherosclerotic plaques of mice and rabbits. Combined dual-target imaging with ^18^F-Macroflor PET and MPO-Gd MRI further revealed macrophage expansion along with a concomitant shift towards pro-reparative phenotypes with reduced MPO production.^22^

#### CD45-PET

CD45 (leukocyte common antigen) is a transmembrane protein tyrosine phosphatase that is expressed on all leukocytes, where it plays a critical role in regulating cell signalling and function. Its specificity to immune cells and abundant expression across various leukocyte subtypes make CD45 a valuable target for imaging inflammation, offering potential clinical applications in multiple inflammatory conditions. Using a CD45-targeting nanobody conjugated with ^89^Zirconium (^89^Zr), Farid *et al.* developed a novel CD45-PET tracer able to longitudinally track inflammation severity in various disease models such as acute respiratory distress syndrome, inflammatory bowel disease, and graft-vs.-host disease,^23^ and demonstrated higher specificity and localized uptake compared to ^18^F-FDG-PET.^24^ It would be of high interest to explore the potential for imaging post-MI inflammation using CD45-PET to visualize leukocyte accumulation in the infarct regions.

#### Chemokine receptor-targeting PET

Chemokine receptors, such as CXCR4 and CCR2, are transmembrane chemokine G-protein-coupled receptors ubiquitously expressed on leukocytes. They are responsible for mediating cell trafficking during inflammation, and play a significant role in leukocyte recruitment into the infarct region. Jujo *et al*. showed that blockade of CXCR4 with small molecule antagonist AMD3100 stimulates neovascularization and functional recovery after MI by enhancing the mobilization of bone marrow–derived endothelial progenitor cells (EPCs).^50^ Single-dose injection of AMD in mice increased EPC production and accumulation at the infarct border, resulting in reduced fibrosis and improved cardiac function. Wang *et al*. demonstrated that POL5551, a peptidic macrocycle CXCR4 antagonist, significantly reduced infarct size and improved left ventricular ejection fraction in mice and pigs. This was associated with increased angiogenesis and reduced inflammatory gene expression in macrophages via regulatory T-cell (Treg) activation.^51^ The clinical benefit of CXCR4-targeted anti-inflammatory treatments may vary depending on the individual baseline CXCR4 expression level in the target tissue and haematopoietic organs.^25^ Hence, a CXCR4-specific imaging probe is crucial for accurate evaluation of treatment efficacy and feasibility.

Gallium^68^ (^68^Ga)-pentixafor is a highly-specific CXCR4-targeting tracer for PET, that has been introduced for imaging tumours and lymphoproliferative disease. In mice (*n* = 53), Thackeray *et al*. showed that ^68^Ga-pentixafor could quantify CXCR4 upregulation in the infarct, peaking at 3 days (infarct/remote [I/R] ratio 1.5 ± 0.2 day 3 vs. 1.2 ± 0.2 day 7, *P* = 0.03).^25^ This corresponded with a flow cytometry-based peak of CD45^+^ leukocytes, and immunohistochemical detection of CD68^+^ macrophages and Ly6G^+^ granulocytes. Furthermore, ^68^Ga-pentixafor was able to identify significant CXCR4 reduction with AMD3100 treatment at 3 days (I/R ratio 1.2 ± 0.2, *P* = 0.05 vs. untreated), persisting up to 7 days (I/R ratio 1.0 ± 0.1, *P* = 0.01 vs. untreated). In MI patients, early ^68^Ga-pentixafor PET signal intensity (3–5 days post-MI) was associated with reduced cardiac function (7.0 ± 2.8 months) (*r* = −0.41, *P* = 0.03), and increased risk of future MACEs.^26,27^

CXCR4 can also be used as a marker for T-cell responses. Rieckmann *et al.* demonstrated that T cells may play a significant reparative role in the myocardium, as the presence of cardiac myosin heavy chain α (MYHA)-derived antigens drives their differentiation into regulatory T cells that suppress inflammation and promote collagen deposition.^28^ While T cells constitute <15% of CXCR4^+^ cells in the heart, they account for >90% of CXCR4^+^ cells in the heart-draining mediastinal lymph nodes (medLNs), making CXCR4 an effective target for visualizing T-cell activity in those areas. Using ^68^Ga-pentixafor PET/CT, the authors found that patients showed significantly increased CXCR4 expression in medLNs within 7 days after MI compared to controls (SUV_max_ 2.7 ± 0.9 vs. 1.9 ± 0.3, *P* < 0.001). Notably, higher medLN CXCR4 signals correlated with improved outcomes, with reduced myocardial necrosis on follow-up cardiac MRI at 4 months (*r* = −0.51, *P* = 0.06).

**Figure 3:**
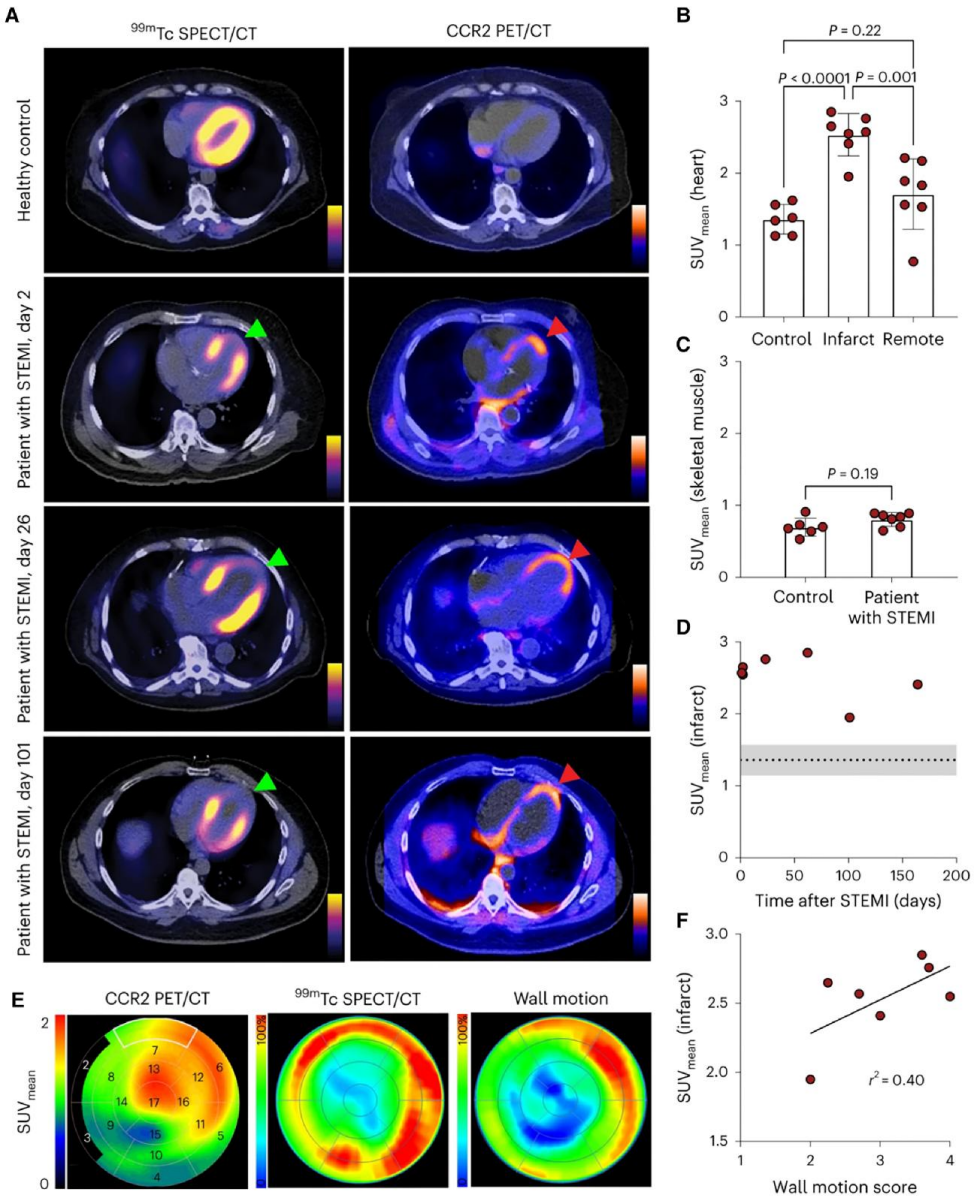
Visualizing myocardial inflammation using ^64^Cu-DOTA-ECL1i PET/CT. *A*) Tc-99m SPECT/CT and ^64^Cu-DOTA-ECL1i PET/CT images from healthy controls and STEMI patients. Regions with increased ^64^Cu-DOTA-ECL1i uptake strongly correlated with infarct areas of reduced perfusion in Tc-99m SPECT/CT (red and green arrowheads). *B, C*) ^64^Cu-DOTA-ECL1i signal intensity (SUV_mean_) was significantly increased in the infarct myocardium of STEMI patients compared to healthy controls. No significant difference in intensity was observed in the skeletal muscle. *D*) ^64^Cu-DOTA-ECL1i signal as a function of time after STEMI (days). Each data point represents an individual STEMI patient, while the line and grey zones represent the mean value of healthy controls with SD. *E*) 17-segment model polar maps representing areas of perfusion and myocardial wall motion from Tc-99m SPECT/CT. *F*) Linear regression analysis showed a significant association between ^64^Cu-DOTA-ECL1i uptake and wall motion score (r^2^ = 0.40, *P* = 0.03). Reproduced from Lavine *et al.*, copyright permission obtained from Springer Nature (CC-BY-NC).^29^

CCR2 is another chemokine receptor highly expressed in macrophages. Lavine *et al.* developed a novel CCR2-targeting probe, DOTA-ECL1i, that can be radiolabelled with Gallium^68^ or Copper^64^ (^68^Ga/^64^Cu-DOTA-ECL1i) to visualize macrophage activity using PET/CT.^29^ In a trial of seven STEMI patients, ^64^Cu-DOTA-ECL1i showed significantly increased uptake in the myocardium compared to healthy controls (SUV_mean_ 2.53 ± 0.3 vs. 1.36 ± 0.2, *P* < 0.001), which was localized to infarct regions with reduced perfusion and myocardial wall motion according to Tc-99m-SPECT/CT (*Figure 3*).

Using a combination of ^64^Cu-DOTA-ECL1i and a ^89^Zr-labelled CD11b-targeting nanotracer, Maier *et al.* characterized myeloid cell infiltration in the infarct myocardium using PET imaging.^30^ In mice with surgically induced MI by either ischaemia–reperfusion (IR) or permanent occlusion (PO), significantly increased uptake of both tracers was observed in the infarct regions, which was validated by *ex vivo* gamma counting. This corresponded with flow cytometry analysis showing increased populations of neutrophils, Ly6C^hi^ monocytes, and macrophages. Subsequent MRI measurements confirmed a strong inverse correlation between the extent of myeloid cell infiltration and left ventricular ejection fraction (*P* < 0.001).

#### TSPO PET

*The translocator protein* (TSPO) is a mitochondrial protein expressed during inflammatory responses. It is particularly upregulated in activated microglia and macrophages, where it modulates production of pro-inflammatory cytokines and ROS, and represents a potential imaging target for inflammation in the myocardium as well as brain tissue. Using a TSPO-targeting PET tracer ^18^F-GE180, Thackeray *et al.* performed longitudinal tracking of macrophage and microglial activation by serial imaging.^31^ Elevated TSPO signal was observed in infarct mice (%injected dose/g 8.0 ± 1.6 vs. 4.8 ± 0.9, *P* < 0.001), localizing to areas of CD68^+^ macrophage accumulation. Pro-inflammatory macrophages showed significantly higher uptake than anti-inflammatory macrophages (*P* < 0.001), highlighting the tracer’s specificity for pro-inflammatory processes. Early TSPO signal at 1 week post-MI predicted subsequent left ventricular remodelling at 8 weeks (*r*_partial_ = −0.687, *P* = 0.001). In parallel, brain TSPO signal was also elevated (*P* < 0.017), localizing to activated microglia. Following a transient decline in uptake at 4 weeks, the myocardial signal rose again at 8 weeks (*P* < 0.001). Notably, this late signal had shifted from the infarct region to the remote myocardium, suggesting ongoing mitochondrial dysfunction and adverse remodelling. This was accompanied by an elevated signal uptake in the brain tissue (*P* = 0.005), indicating persistent neuroinflammation. ACE inhibitor treatment reduced acute inflammation in both the heart (*P* = 0.003) and the brain (*P* = 0.06), and improved late cardiac function (*P* = 0.05). Similar results were obtained in STEMI patients, with elevated TSPO uptake in the infarct region (*P* = 0.017) and evidence of concomitant neuroinflammation.

It has previously been observed that cognitive impairment is more frequent in patients with a history of MI.^52^ Additionally, the extent of coronary artery disease has been linked to neurodegenerative disorders such as Alzheimer’s,^53^ potentially through mechanisms involving neuroinflammation. This study demonstrated that TSPO-targeted imaging not only detects early post-infarct myocardial inflammation, whose severity predicts later adverse remodelling, but also identifies microglia activation as a driver of neuroinflammation, which coincides with both the acute phase of myocardial inflammation and the chronic phrase of HF. These findings suggest a mechanistic link between sustained myocardial inflammation and persistent neuroinflammation that may explain the accelerated cognitive decline associated with heart disease, and highlight new avenues for therapeutic intervention.

### Myofibroblast-targeting agents for the proliferative phase

Myofibroblasts deposit collagen and elastin for fibrogenesis. Although this is important for early myocardial repair and scar maintenance, excessive fibrotic activity impairs ventricular contractility and function. Imaging fibroblast activity not only serves as a potential predictor of myocardial healing, but may also help identify a time window where excessive ongoing fibrosis can be reversed with anti-fibrotic treatments targeting fibroblast activation pathways.

#### Fibroblast activation protein inhibitors

Fibroblast activation protein inhibitors (FAPIs) bind to fibroblast activation protein (FAP), a membrane-bound serine protease expressed by activated fibroblasts. ^68^Ga-labelled FAPIs have previously been used in conjunction with serial ^18^F-FDG-PET for staging of various malignancies such as head, neck, and abdominal tumours. FAPI-04, a ^68^Ga-labelled FAPI, was first trialled for post-infarct remodelling in mice (*n* = 20).^32^ PET/CT images showed that FAPI-04 uptake peaked on Day 6, and high uptake corresponded to areas of low metabolism in ^18^F-FDG-PET. Notohamiprodjo *et al*. further observed that combined use of FAPI-04 PET and LGE-MRI could identify ongoing myofibroblast migration to remote healthy myocardium, an early and potentially reversible manifestation of adverse remodelling that cannot be detected using conventional LGE-MRI alone.^33^

**Figure 4:**
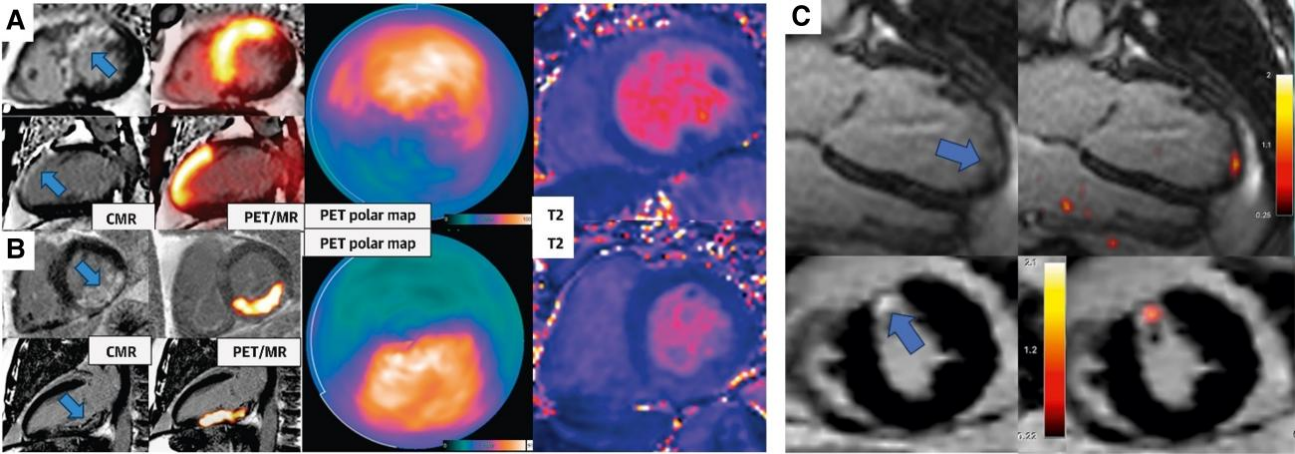
Visualizing acute and chronic fibroblast activity in the infarct and remote myocardium using ^68^Ga-FAPI-46 PET/MRI. Short-axis and 2-chamber PET and LGE-MRI overlay images at *A*) 23 days and *B*) 3 days post-MI. Intense FAPI-46 activity was observed in the infarct zone coinciding with areas of late gadolinium enhancement (blue arrows), and also extending into the peri-infarct regions. *C*) Long-axis and short-axis PET and LGE-MRI images, taken at 2.5 years (top) and 20 years (bottom) post-MI respectively. Low levels of persistent fibroblast activation was detected by FAPI-46 that was confined within areas of chronic infarction. Reproduced from Barton *et al.*, open access under Creative Commons CC-BY 4.0.^34^

Barton *et al*. conducted the first longitudinal study to investigate the long-term distribution and time course of fibroblast activity in MI patients (*n* = 40) (*Figure 4*).^34^ Using a novel ^68^Ga-labelled FAPI-46, they established strong positive association between high baseline uptake and subsequent left ventricular end-diastolic volumes (*P* < 0.001) and scar burden (*P* < 0.001) at 12 months. Further, they showed that intense fibroblast activation could persist up to 3 months, with low levels detectable even years later.

#### Integrin-targeting agents

Integrins, such as α_v_β_3_, are transmembrane cell surface receptors that facilitate cell migration, proliferation, and interactions with the ECM. They are expressed by vascular endothelial cells in states of angiogenesis early after MI, and are markedly upregulated on activated myofibroblasts during the proliferative phase to promote collagen production and deposition. α_v_β_3_ can be targeted using short peptide sequences such as arginine–glycine–aspartic acid (RGD)-peptides. This was first discovered in 1991 by Aumailley *et al*., who reported highly selective binding of cyclic pentapeptides containing the RGD sequence to α_v_β_3_.^54^ Since then, a range of RGD-containing agents have been developed for targeting tumorigenesis, angiogenesis, and fibroblasts. van den Borne *et al*. proposed a Tc-99m-labelled Cy5.5-RGD imaging peptide (CRIP), providing clear characterization of myofibroblasts with SPECT in mice (*n* = 41).^35^ Uptake peaked at 2 weeks post-injection (%injected dose/g 2.75 ± 0.46%), followed by 4 (2.26 ± 0.09%) and 12 weeks (1.74 ± 0.24%) compared with controls (0.59 ± 0.19%). Furthermore, CRIP was able to quantitatively monitor the attenuation of fibroblast activity by RAAS-targeting neurohumoral antagonist treatments such as ACE inhibitors and aldosterone antagonists. This was associated with a significant reduction in wall thinning, fibrosis, and collagen deposition by histological staining.

RGD-peptides can also be combined with ^18^F or ^68^Ga and imaged using PET. These include ^18^F-galacto-RGD, ^18^F-fluciclatide, ^68^Ga-NOTA-RGD, and ^68^Ga-NODAGA-RGD, which have shown promising results in pre-clinical and clinical trials.^36^ In a study on 21 STEMI patients, ^18^F-fluciclatide uptake significantly increased in areas of regional wall hypokinesia (*P* < 0.001).^37^ Although there was no significant correlation with infarct size or inflammation, early uptake at 2 weeks post-MI was associated with functional recovery at 9 months. Similarly, in a 2024 study of 31 patients, ^68^Ga-NODAGA-RGD identified increased α_v_β_3_ expression in the infarct compared with remote myocardium (*P* < 0.001), and early uptake (7.7 ± 3.8 days) predicted improvements in global left ventricular function at 6 months (*P* = 0.002).^38^

#### α7nAChR-targeting agents

**Figure 5:**
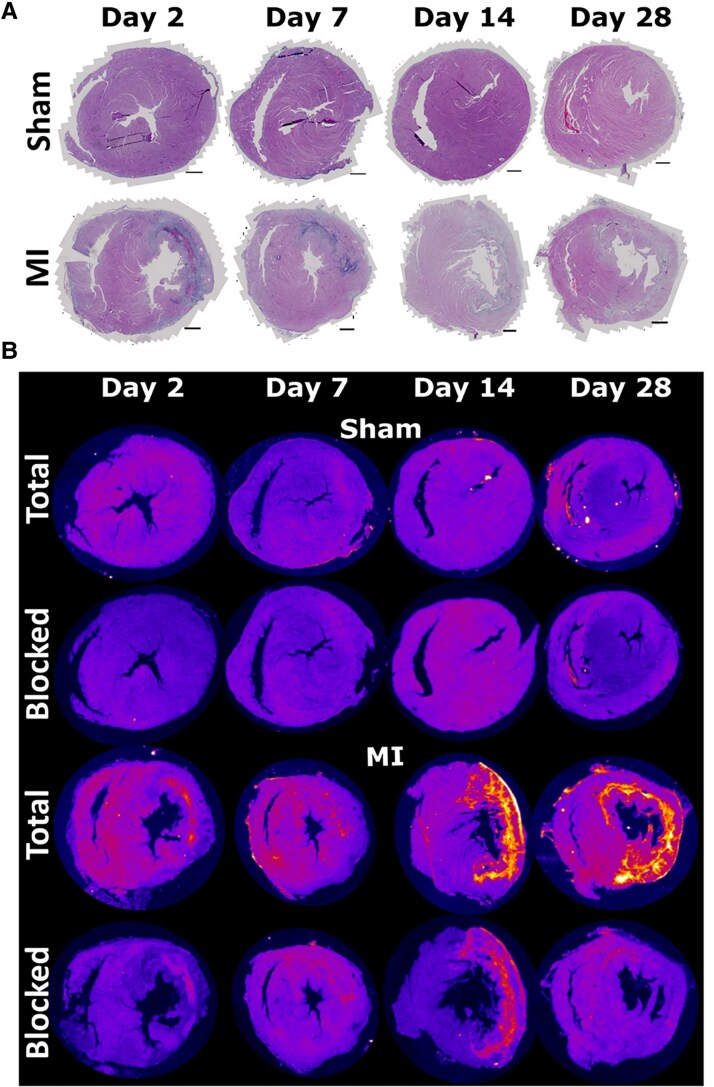
Quantification of ɑ7nAChR expression in the infarct myocardium with NS14490-PET. *A*) Histological H&E staining of non-infarcted (sham) and MI hearts from day 2 to day 28. Scale bar = 1 mm. *B*) Corresponding NS14490-PET images. Enhanced uptake was observed on days 14 to 28 in the infarct regions in the left ventricle of MI mice. Reproduced from Reid *et al.*, open access under Creaetive Commons CC-BY 4.0.^39^

The alpha-7 nicotinic acetylcholine receptor (ɑ7nAChR) is a pentameric-ligand-gated ion channel expressed by endothelial cells, vascular smooth muscle cells, immune cells, and fibroblasts. It plays an important regulator of angiogenesis and fibrosis following the inflammatory phase, and stimulates the polarization of macrophages to the anti-inflammatory phenotype. Reid *et al.* investigated its utility for assessing angiogenesis and anti-inflammatory activity, using a ɑ7nAChR-targeting tritium-labelled PET tracer [^3^H]-NS14490^39^ (*Figure 5*). In MI rats, specific binding in the infarct increased from day 14 post-MI (33.8 ± 14.1 µCi/g, *P* ≤ 0.01 vs. sham), peaking at day 28 (48.9 ± 5.1 µCi/g, *P* ≤ 0.0001 vs. sham). While NS14490 imaging displayed patterns of vessel formation, macrophage infiltration, and fibrovascular encapsulation in mouse models of inflammatory angiogenesis, this was not the case in the MI model, where uptake was more strongly associated with ECM production, which coincided with ɑ7nAChR upregulation in mouse cardiac fibroblasts under hypoxic conditions. This suggests that while ɑ7nAChR is involved in multiple processes of myocardial repair, it is most closely associated with fibroblast activity following MI.

### Collagen and elastin-targeting agents for the maturation phase

Several gadolinium-containing agents have been developed to target scar ECM proteins during the maturation phase. By enabling quantification of intra-scar collagen and elastin content *in vivo*, they aim to provide clear and non-invasive characterization of fibrosis, and could potentially be used for monitoring collagen and elastin-targeting treatments.

#### Collagen-targeting EP-3533

EP-3533 is a gadolinium agent that targets type I collagen. Unlike Gd-DTPA, EP-3533 contains three gadolinium ions per molecule. This not only enhances the relaxivity of each molecule as a whole, but also that of each gadolinium ion itself, resulting in an overall 15-fold increase in sensitivity.^40^ Additionally, direct binding to collagen provides more persistent contrast than Gd-DTPA. In mice (*n* = 8), Helm *et al*. demonstrated EP-3533’s ability to provide accurate delineation of the infarct scar. Serial imaging at 5, 20, and 35 min post-injection showed sharp and persistent contrast, with significantly longer washout time compared with Gd-DTPA (mean 194.9 ± 116.8 min vs. 25.5 ± 4.2 min, *P* < 0.05).^40^

### ^68^Ga-CBP8

^68^Ga-Collagen Binding Probe #8 (^68^Ga-BP8) is a PET tracer that also targets type I collagen, providing direct quantification of collagen deposition with rapid renal clearance. In patients with idiopathic pulmonary fibrosis (IPF), ^68^Ga-BP8 was safe and well-tolerated.^41^ Increased tracer signal was observed not only within areas of known fibrosis, but also in regions where active or recent collagen synthesis was not yet visible on conventional CT imaging. While its efficacy has yet to be tested in other non-IPF pathologies, there is strong potential for ^68^Ga-BP8 to be applied in longitudinal monitoring of post-infarct myocardial fibrosis and detection of early responses to anti-fibrotic treatments.

 

#### Elastin-targeting ESMA

Alongside collagen, elastin is a key component of the scar ECM, maintaining the elasticity of the ventricular wall. Low elastin content causes myocardial stiffening and reduced contractility.

**Figure 6:**
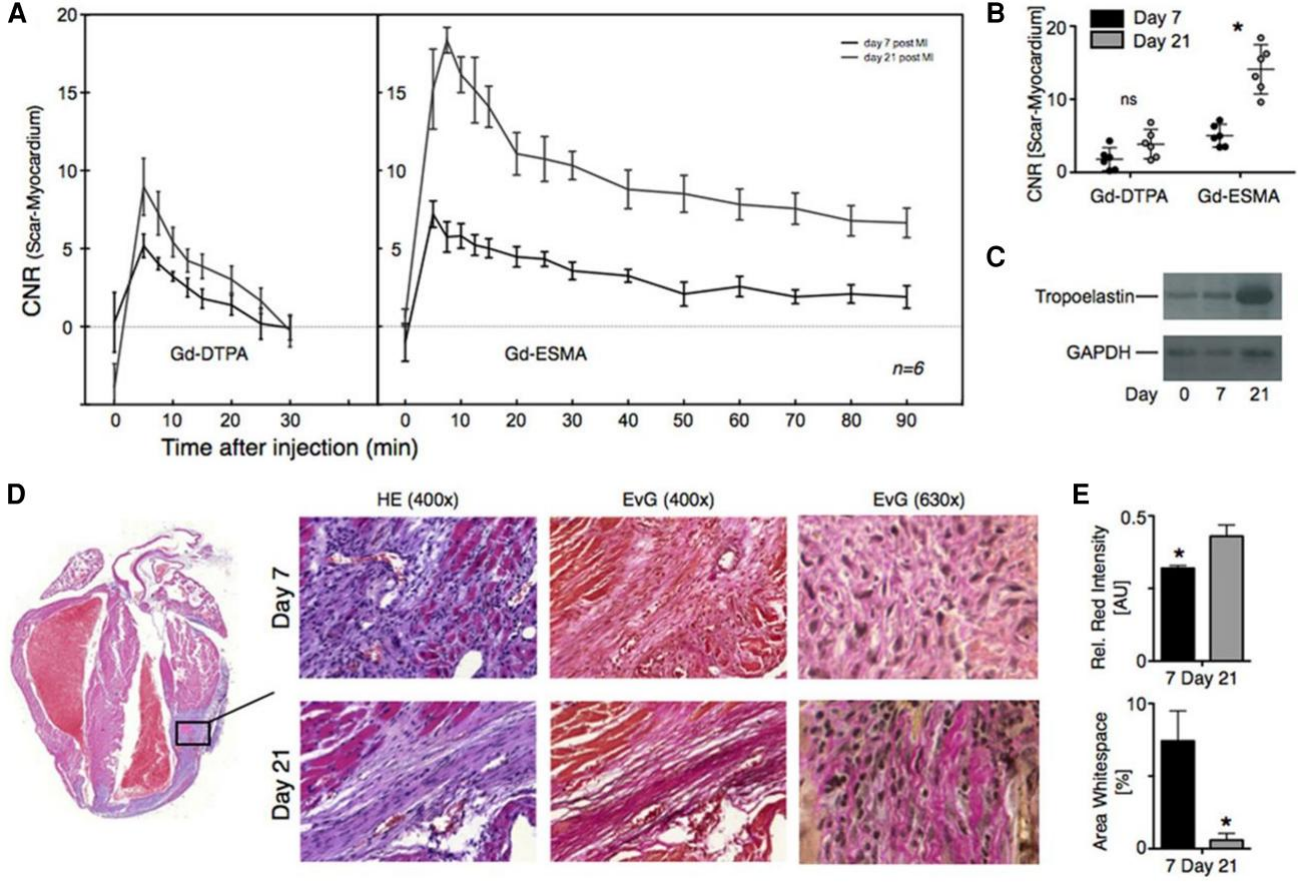
Quantification of elastin with MRI using Gd-ESMA vs. Gd-DTPA in late adverse modelling. *A*) Consecutive imaging on day 7 and day 21 with Gd-DTPA vs. Gd-ESMA. While no significant difference in uptake of Gd-DTPA was observed between day 7 and day 21, uptake of Gd-ESMA was significantly increased on day 21. Furthermore, the Gd-ESMA signal was persistent up to 90 min after injection, while Gd-DTPA was quickly flushed out 30 min after injection. *B*) Dot plot diagram of contrast-to-noise ratio (CNR) values (mean and 95% confidence interval) on day 7 vs. day 21 post-MI. While CNR values did not differ significantly for Gd-DTPA, they were significantly higher on day 21 compared to day 7 for Gd-ESMA (*P* = 0.0032). This corresponded with increase in elastin deposition within the infarct as verified by ex vivo *C*) Western blot analysis, and *D, E*) histological haematoxylin and eosin (HE) and elastica van Gieson (EvG) staining. *P < 0.05. Reproduced from Wildgruber *et al.*, copyright permission obtained from Wolters Kluwer Health Inc. (CC-BY-NC).^42^

Gd-ESMA is a gadolinium-containing probe that targets elastin. Previously known for imaging atherosclerotic vessel walls, its applicability for post-infarct remodelling has recently been studied. Similar to EP-3533 with collagen, Gd-ESMA’s strong affinity to elastin allows it to generate more persistent contrast compared with Gd-DTPA. In mice (*n* = 40), Wildgruber *et al*. demonstrated that while Gd-DTPA was quickly washed out from the infarct shortly after injection, Gd-ESMA showed prolonged enhancement of the infarct scar, and could accurately quantify the increase in elastin deposition up to 21 days post-MI^42^ (*Figure 6*). Similarly, Elkenhans *et al*. showed that ESMA enabled clear visualization of infarct scar in mice up to 30 days post-MI, with strong correlation to histological staining of collagen types I and III.^43^

## Discussion and future prospects

Molecular tracers have shown great promise for assessing post-infarction remodelling *in vivo*. The ability to bind specific molecular targets makes it possible to directly visualize key stages of remodelling, as well as quantifying cellular accumulation and molecular processes through T1/T2* mapping studies (for MRI) or standardized uptake value quantification (for PET). Their high specificity enables selective accumulation at the infarct, producing strong persistent signals with minimal background uptake. In both animal and early clinical studies, molecular imaging was able to longitudinally track the evolution of myocardial repair and remodelling, and quantitatively monitor treatment effects over time. Probes targeting inflammatory cells and fibroblasts have been able to quantify changes in myocardial inflammation and fibrosis following conventional treatments, such as statins, aldosterone antagonists, and ACE-inhibitors, as well as novel targeted treatments. The ability to non-invasively monitor the precise molecular and cellular pathways of remodelling could ultimately be utilized by clinicians to provide an accurate assessment of disease progression and prognosis, and enable the provision of personalized treatment. As these techniques mature, molecular tracers may supplement, or even replace, current imaging approaches for guiding therapy decisions in CVD.

Nevertheless, large-scale clinical trials are required to confirm their clinical efficacy. To date, most molecular MRI tracers remain limited to animal studies, while molecular PET agents have only been tested in small-scale clinical trials. Clinical translation is a costly and time-consuming process: molecular probes are expensive to mass-produce, and some such as SPIONs require long waiting times between injection and imaging. Additionally, the higher dosage required in humans compared to smaller animal models warrants the need for further studies to evaluate their safety and viability, particularly in patients with co-morbidities such as hypertension, hypercholesterolaemia, and diabetes. This includes long-term toxicity studies on other organs.

Alongside technical and logistical challenges, continuous strides can be made to broaden the range of molecular targets. For example, the majority of macrophage-targeting tracers do not distinguish between pro-inflammatory and anti-inflammatory subsets, highlighting the need for more specific markers to differentiate between inflammatory vs. pro-reparative cellular processes. Recent studies have also shed light on the role of the adaptive immune system, particularly B cells and T cells, in orchestrating the post-infarct inflammatory response. Early clinical studies, such as the LILACS^55^ and RITA-MI trials,^56^ have explored the use of targeted immunomodulatory therapies to suppress post-MI inflammation by regulating specific B-cell and T-cell subsets, opening the door to new potential targets to monitor these treatments.

Combined multi-tracer imaging may help provide more detailed characterization of the interactions between different cellular pathways involved in inflammation and healing. For example, Hess *et al.* showed that systemic macrophage depletion in mice reduced uptake of TSPO-targeting ^18^F-GE180 post-MI due to lower CD68^+^ macrophage populations, while increasing CXCR4^+^ neutrophil recruitment evaluated by ^68^Ga-pentixafor.^57^ Interest in such multi-tracer imaging approaches has emerged in recent years with the concept of the *immune-fibrosis axis*, in which early immune responses from the inflammatory phase shape downstream fibroblast fates, matrix composition, and scar maturation, thereby supporting the rationale for combined imaging of both inflammatory and fibrotic processes. In mice, Ramos *et al.* demonstrated that combined simultaneous MRI with ^19^F-perfluorocarbon (PFC) nanotracers for inflammatory cell recruitment and Gd-ESMA for elastin predicted favourable post-MI remodelling significantly better than each of the tracers alone.^58^ Likewise, studies exploring sequential multi-tracer PET approaches, such as ^68^Ga-DOTA-ECL1i for inflammatory cells followed by ^68^Ga-FAPI-46 for subsequent fibroblast activation,^59^ have highlighted the value of temporal inflammation-fibrosis imaging and the importance standardized quantification protocols for cross-centre reproducibility.

The use of hybrid imaging PET/MRI further integrates the detailed assessment of cardiac function and tissue viability from MRI, with the cell-specificity and sensitivity from molecular PET imaging.^49^ With the advent of total-body PET scanners, extending the axis field-of-view to encompass the entire body, tracers can also be used to visualize post-MI inflammatory mechanisms on a systemic level, linking key interactions between the cardiovascular system and other organs such as the brain^31^ and spleen^60^; whilst at the same time enabling lower radiation and quicker scans with higher image resolution, and allowing delayed acquisitions to improve image contrast.^60,61^

Thus, whilst significant challenges remain, molecular imaging holds great potential for translation to clinical practice. Ongoing developments in tracer design and imaging techniques will pave the way for its integration into patient care, with the ultimate goal to serve as an adjunct for disease stratification, clinical decision-making and provision of targeted treatments.

## Conclusion and learning points

Post-MI remodelling and its associated complications remain a significant cause of mortality and reduced quality of life following infarction. Molecular imaging represents a promising approach for visualizing specific cellular processes underlying adverse remodelling, to improve prognostic accuracy and individualized treatment. PET and MRI molecular probes have demonstrated strong potential in pre-clinical and early clinical trials for stratifying stages of remodelling, and monitoring the therapeutic effects of targeted treatments. Continued technical advancements and larger-scale clinical trials are warranted to improve sensitivity and test the clinical applicability of molecular tracers. Nevertheless, there is potential for molecular imaging to become a powerful tool for improving patient outcomes.

## Data Availability

No new data were generated or analysed in support of this research.

## Lead author biography

Pak Yin Lam is a medical student at King’s College London and a Master’s student in Cardiovascular Medicine at the University of Cambridge, where he joined the lab of Professor Ziad Mallat at the Heart & Lung Research Institute to study how the adaptive immune system drives inflammatory processes in atherosclerosis. His particular research interests include the pathophysiology of inflammation in heart disease, as well as the assessment and management of post-infarct myocardial injury.
